# Dichloridotris(2-methyl-1*H*-imidazole-κ*N* ^3^)cadmium

**DOI:** 10.1107/S160053681201104X

**Published:** 2012-03-17

**Authors:** Run-Qiang Zhu

**Affiliations:** a Ordered Matter Science Research Center, College of Chemistry and Chemical Engineering, Southeast University, Nanjing 211189, People’s Republic of China

## Abstract

In the title compound, [CdCl_2_(C_4_H_6_N_2_)_3_], the Cd^II^ atom displays a penta­coordinate CdN_3_Cl_2_ coordination geometry, being coordinated by an N atom of three 2-methyl­imidazole ligands and two Cl atoms. In the crystal, the mononuclear complexes are linked by N—H⋯Cl hydrogen bonds into a two-dimensional network in the *ab* plane.

## Related literature

For general background to ferroelectric metal-organic frameworks, see: Fu *et al.* (2009 ▶); Ye *et al.* (2006 ▶); Zhang *et al.* (2008 ▶, 2010 ▶).
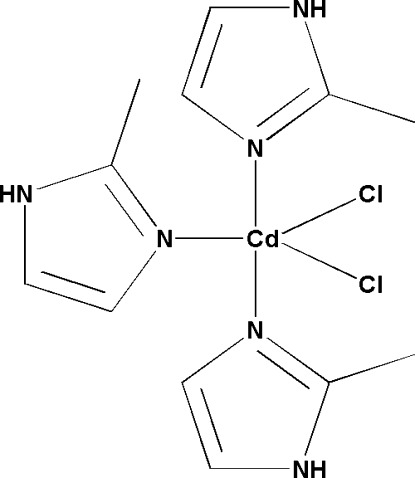


## Experimental

### 

#### Crystal data

[CdCl_2_(C_4_H_6_N_2_)_3_]
*M* *_r_* = 429.62
Monoclinic, 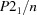

*a* = 8.2983 (17) Å
*b* = 15.069 (3) Å
*c* = 14.266 (3) Å
β = 104.76 (3)°
*V* = 1725.1 (6) Å^3^
*Z* = 4
Mo *K*α radiation
μ = 1.58 mm^−1^
*T* = 293 K
0.28 × 0.26 × 0.20 mm

#### Data collection

Rigaku SCXmini diffractometer
Absorption correction: multi-scan (*CrystalClear*; Rigaku, 2005 ▶) *T*_min_ = 0.649, *T*_max_ = 0.729
17235 measured reflections
3919 independent reflections
3608 reflections with *I* > 2σ(*I*)
*R*_int_ = 0.048
2 standard reflections every 150 reflections intensity decay: none

#### Refinement

*R*[*F* ^2^ > 2σ(*F* ^2^)] = 0.027
*wR*(*F* ^2^) = 0.063
*S* = 1.12
3919 reflections
191 parameters
H-atom parameters constrained
Δρ_max_ = 0.36 e Å^−3^
Δρ_min_ = −0.92 e Å^−3^

### 

Data collection: *CrystalClear* (Rigaku, 2005 ▶); cell refinement: *CrystalClear* (Rigaku, 2005 ▶); data reduction: *CrystalClear* (Rigaku, 2005 ▶); program(s) used to solve structure: *SHELXS97* (Sheldrick, 2008 ▶); program(s) used to refine structure: *SHELXL97* (Sheldrick, 2008 ▶); molecular graphics: *DIAMOND* (Brandenburg & Putz, 2005 ▶); software used to prepare material for publication: *SHELXL97* (Sheldrick, 2008 ▶).

## Supplementary Material

Crystal structure: contains datablock(s) I, global. DOI: 10.1107/S160053681201104X/su2390sup1.cif

Structure factors: contains datablock(s) I. DOI: 10.1107/S160053681201104X/su2390Isup2.hkl

Additional supplementary materials:  crystallographic information; 3D view; checkCIF report

## Figures and Tables

**Table 1:** Hydrogen-bond geometry (Å, °)

*D*—H⋯*A*	*D*—H	H⋯*A*	*D*⋯*A*	*D*—H⋯*A*
N6—H6*A*⋯Cl1^i^	0.86	2.60	3.387 (2)	152
N4—H4*B*⋯Cl2^ii^	0.86	2.59	3.382 (2)	154
N2—H2*A*⋯Cl1^iii^	0.86	2.45	3.253 (2)	156

Symmetry codes: (i) 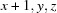; (ii) 
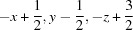
; (iii) 
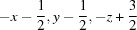
.

**Fig. 1.**
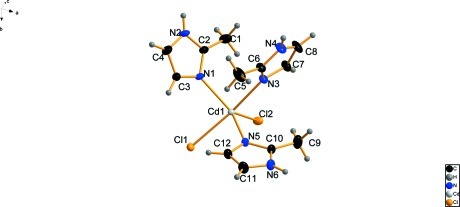
A view of the moolecular structure of the title compound, with the atom numbering. The displacement ellipsoids are drawn at the 30% probability level.

**Fig. 2.**
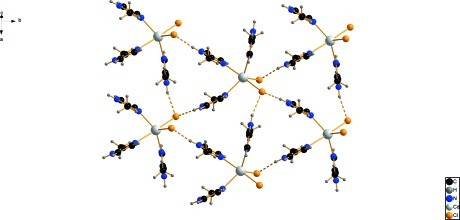
A view along the c axis of the two-dimensional hydrogen bonded network of the title compound. The N-H···Cl bonds are shown as dashed lines; see Table 1 for details.

## supplementary crystallographic information

### Comment

As part of our ongoing studies of potential ferroelectric phase change materials we have determined the structures of several chromium complexes and examined the changes in their dielectric constants with temperature, which is the usual method for detecting such behaviour, as shown by (Fu *et al.*, 2009; Ye *et al.*, 2006; Zhang *et al.*, 2008; Zhang *et al.*, 2010). The dielectric constant of the title cadmium(II) compound indicates the onset of a ferroelectric phase change over the range 80–298 K.

As shown in Fig. 1, the Cd^II^ ion adopts a pentacoordinate geometry and is coordinated by an N atom from three independent 2-methyl-imidazole ligands and by two Cl atoms. The bond length of the middle Cd1–N3 bond is 2.357 (3) Å, which is longer than the other two Cd—N bond lengths [Cd1—N1= 2.276 (3) Å and Cd1—N5= 2.289 (3) Å].

In the crystal, the mononuclear complexes are linked by N–H···Cl hydrogen bonds to form a two-dimensional network in the ab plane (Fig. 2 and Table 1).

### Experimental

An aqueous solution of 2-methyl-imidazole (1.64 g, 20 mmol) and hydrochloric acid (10 ml) was treated with CdCl_2_ (1.35 g, 10 mmol). After the mixture had been stirred for a few minutes, it was left to stans for a few days. Slow evaporation of the solution yielded colourless X-ray quality crystals.

### Refinement

The NH and C-bound H-atoms were included in calculated positions and treated as riding atoms: N-H = 0.86 Å, C-H = 0.93 and 0.96 Å for CH, and CH_3_ H-atoms, respectively, with U_iso_(H) = k × U_eq_(N,C), where k = 1.5 for CH_3_ H-atoms and = 1.2 for other H-atoms.

### Crystal data

**Table:**

[CdCl_2_(C_4_H_6_N_2_)_3_]	*F*(000) = 856
*M**_r_* = 429.62	*D*_x_ = 1.654 Mg m^−^^3^
Monoclinic, *P*2_1_/*n*	Mo *K*α radiation, λ = 0.71073 Å
Hall symbol: -P 2yn	Cell parameters from 3960 reflections
*a* = 8.2983 (17) Å	θ = 2.3–27.5°
*b* = 15.069 (3) Å	µ = 1.58 mm^−^^1^
*c* = 14.266 (3) Å	*T* = 293 K
β = 104.76 (3)°	Block, colourless
*V* = 1725.1 (6) Å^3^	0.28 × 0.26 × 0.20 mm
*Z* = 4	

### Data collection

**Table:**

Rigaku SCXmini diffractometer	3608 reflections with *I* > 2σ(*I*)
Radiation source: fine-focus sealed tube	*R*_int_ = 0.048
Graphite monochromator	θ_max_ = 27.5°, θ_min_ = 3.1°
CCD_Profile_fitting scans	*h* = −10→10
Absorption correction: multi-scan (*CrystalClear*; Rigaku, 2005)	*k* = −19→19
*T*_min_ = 0.649, *T*_max_ = 0.729	*l* = −18→18
17235 measured reflections	2 standard reflections every 150 reflections
3919 independent reflections	intensity decay: none

### Refinement

**Table:**

Refinement on *F*^2^	Secondary atom site location: difference Fourier map
Least-squares matrix: full	Hydrogen site location: inferred from neighbouring sites
*R*[*F*^2^ > 2σ(*F*^2^)] = 0.027	H-atom parameters constrained
*wR*(*F*^2^) = 0.063	*w* = 1/[σ^2^(*F*_o_^2^) + (0.0191*P*)^2^ + 0.8231*P*] where *P* = (*F*_o_^2^ + 2*F*_c_^2^)/3
*S* = 1.12	(Δ/σ)_max_ = 0.002
3919 reflections	Δρ_max_ = 0.36 e Å^−^^3^
191 parameters	Δρ_min_ = −0.92 e Å^−^^3^
0 restraints	Extinction correction: *SHELXL97* (Sheldrick, 2008), Fc^*^=kFc[1+0.001xFc^2^λ^3^/sin(2θ)]^-1/4^
Primary atom site location: structure-invariant direct methods	Extinction coefficient: 0.0486 (9)

### Special details

**Table:**

Geometry. All e.s.d.'s (except the e.s.d. in the dihedral angle between two l.s. planes) are estimated using the full covariance matrix. The cell e.s.d.'s are taken into account individually in the estimation of e.s.d.'s in distances, angles and torsion angles; correlations between e.s.d.'s in cell parameters are only used when they are defined by crystal symmetry. An approximate (isotropic) treatment of cell e.s.d.'s is used for estimating e.s.d.'s involving l.s. planes.
Refinement. Refinement of *F*^2^ against ALL reflections. The weighted *R*-factor *wR* and goodness of fit *S* are based on *F*^2^, conventional *R*-factors *R* are based on *F*, with *F* set to zero for negative *F*^2^. The threshold expression of *F*^2^ > σ(*F*^2^) is used only for calculating *R*-factors(gt) *etc*. and is not relevant to the choice of reflections for refinement. *R*-factors based on *F*^2^ are statistically about twice as large as those based on *F*, and *R*- factors based on ALL data will be even larger.

### Fractional atomic coordinates and isotropic or equivalent isotropic displacement parameters (Å^2^)

**Table:**

	*x*	*y*	*z*	*U*_iso_*/*U*_eq_	
C1	−0.1137 (4)	0.0341 (2)	0.8747 (2)	0.0599 (9)	
H1A	−0.0482	0.0869	0.8922	0.090*	
H1B	−0.0430	−0.0171	0.8899	0.090*	
H1C	−0.1975	0.0317	0.9102	0.090*	
C2	−0.1954 (3)	0.03544 (17)	0.76864 (19)	0.0370 (6)	
C3	−0.2860 (3)	0.07277 (18)	0.6168 (2)	0.0416 (6)	
H3A	−0.3038	0.1050	0.5595	0.050*	
C4	−0.3544 (4)	−0.0069 (2)	0.6266 (2)	0.0533 (8)	
H4A	−0.4262	−0.0396	0.5783	0.064*	
C5	0.1447 (5)	0.0007 (2)	0.6192 (2)	0.0643 (10)	
H5A	0.0755	0.0510	0.5950	0.096*	
H5B	0.0777	−0.0520	0.6102	0.096*	
H5C	0.2295	−0.0049	0.5847	0.096*	
C6	0.2248 (3)	0.01344 (17)	0.72517 (18)	0.0366 (6)	
C7	0.3032 (4)	0.06615 (18)	0.8714 (2)	0.0445 (7)	
H7A	0.3141	0.1037	0.9244	0.053*	
C8	0.3793 (4)	−0.0136 (2)	0.8729 (2)	0.0550 (8)	
H8A	0.4512	−0.0409	0.9258	0.066*	
C9	0.4629 (4)	0.2488 (3)	0.7356 (2)	0.0603 (9)	
H9A	0.4012	0.2419	0.7836	0.090*	
H9B	0.5201	0.3047	0.7447	0.090*	
H9C	0.5426	0.2015	0.7418	0.090*	
C10	0.3461 (3)	0.24599 (18)	0.63706 (19)	0.0359 (6)	
C11	0.2578 (3)	0.2484 (2)	0.4767 (2)	0.0496 (8)	
H11A	0.2551	0.2511	0.4112	0.060*	
C12	0.1267 (3)	0.23813 (19)	0.51574 (19)	0.0400 (6)	
H12A	0.0161	0.2333	0.4806	0.048*	
N1	−0.1852 (2)	0.09900 (13)	0.70559 (15)	0.0347 (5)	
N2	−0.2963 (3)	−0.02975 (15)	0.72257 (17)	0.0468 (6)	
H2A	−0.3202	−0.0777	0.7488	0.056*	
N3	0.2063 (3)	0.08299 (13)	0.77834 (15)	0.0341 (5)	
N4	0.3283 (3)	−0.04584 (15)	0.78021 (18)	0.0483 (6)	
H4B	0.3577	−0.0958	0.7605	0.058*	
N5	0.1816 (2)	0.23594 (14)	0.61610 (14)	0.0319 (4)	
N6	0.3953 (3)	0.25409 (17)	0.55365 (16)	0.0443 (6)	
H6A	0.4962	0.2615	0.5498	0.053*	
Cd1	0.021838 (19)	0.201370 (10)	0.720480 (12)	0.02453 (8)	
Cl1	−0.19805 (7)	0.31401 (4)	0.62092 (4)	0.03134 (14)	
Cl2	0.10468 (9)	0.29333 (4)	0.87083 (4)	0.03570 (15)	

### Atomic displacement parameters (Å^2^)

**Table:**

	*U*^11^	*U*^22^	*U*^33^	*U*^12^	*U*^13^	*U*^23^
C1	0.078 (2)	0.0542 (18)	0.0454 (17)	−0.0181 (17)	0.0111 (15)	0.0106 (15)
C2	0.0358 (13)	0.0310 (12)	0.0454 (14)	−0.0048 (10)	0.0125 (11)	0.0063 (11)
C3	0.0385 (14)	0.0369 (14)	0.0437 (15)	−0.0061 (11)	0.0002 (11)	0.0096 (12)
C4	0.0529 (18)	0.0442 (16)	0.0543 (17)	−0.0205 (14)	−0.0020 (14)	0.0016 (14)
C5	0.081 (2)	0.0572 (19)	0.0457 (17)	0.0264 (18)	0.0005 (16)	−0.0192 (16)
C6	0.0410 (14)	0.0294 (12)	0.0389 (13)	0.0107 (11)	0.0096 (11)	−0.0039 (11)
C7	0.0497 (16)	0.0404 (14)	0.0371 (14)	0.0117 (12)	−0.0005 (12)	−0.0032 (12)
C8	0.0583 (19)	0.0482 (17)	0.0493 (17)	0.0240 (15)	−0.0033 (14)	0.0036 (14)
C9	0.0354 (15)	0.097 (3)	0.0478 (17)	−0.0058 (17)	0.0094 (13)	0.0041 (19)
C10	0.0255 (12)	0.0451 (15)	0.0395 (14)	0.0032 (10)	0.0129 (10)	0.0050 (12)
C11	0.0423 (16)	0.074 (2)	0.0365 (15)	0.0036 (15)	0.0170 (12)	0.0099 (15)
C12	0.0305 (13)	0.0520 (16)	0.0368 (14)	0.0021 (12)	0.0075 (10)	0.0060 (13)
N1	0.0347 (11)	0.0240 (10)	0.0451 (12)	−0.0059 (8)	0.0099 (9)	0.0046 (9)
N2	0.0512 (14)	0.0315 (11)	0.0560 (14)	−0.0151 (10)	0.0104 (11)	0.0104 (11)
N3	0.0384 (11)	0.0271 (10)	0.0360 (11)	0.0091 (9)	0.0081 (9)	−0.0033 (9)
N4	0.0541 (15)	0.0296 (11)	0.0577 (15)	0.0193 (10)	0.0076 (12)	−0.0048 (11)
N5	0.0235 (10)	0.0402 (11)	0.0339 (11)	0.0018 (8)	0.0106 (8)	0.0050 (9)
N6	0.0265 (11)	0.0644 (16)	0.0465 (13)	0.0029 (10)	0.0175 (10)	0.0086 (12)
Cd1	0.02507 (11)	0.01824 (11)	0.03208 (12)	0.00024 (6)	0.01061 (7)	−0.00117 (6)
Cl1	0.0265 (3)	0.0266 (3)	0.0400 (3)	0.0036 (2)	0.0068 (2)	0.0002 (2)
Cl2	0.0493 (4)	0.0285 (3)	0.0308 (3)	−0.0028 (2)	0.0130 (3)	−0.0049 (2)

### Geometric parameters (Å, º)

**Table:**

C1—C2	1.492 (4)	C8—H8A	0.9300
C1—H1A	0.9600	C9—C10	1.491 (4)
C1—H1B	0.9600	C9—H9A	0.9600
C1—H1C	0.9600	C9—H9B	0.9600
C2—N1	1.331 (3)	C9—H9C	0.9600
C2—N2	1.348 (3)	C10—N5	1.330 (3)
C3—C4	1.350 (4)	C10—N6	1.358 (3)
C3—N1	1.386 (3)	C11—C12	1.351 (4)
C3—H3A	0.9300	C11—N6	1.370 (3)
C4—N2	1.374 (4)	C11—H11A	0.9300
C4—H4A	0.9300	C12—N5	1.388 (3)
C5—C6	1.501 (4)	C12—H12A	0.9300
C5—H5A	0.9600	N1—Cd1	2.2781 (19)
C5—H5B	0.9600	N2—H2A	0.8600
C5—H5C	0.9600	N3—Cd1	2.359 (2)
C6—N3	1.325 (3)	N4—H4B	0.8600
C6—N4	1.345 (3)	N5—Cd1	2.292 (2)
C7—C8	1.356 (4)	N6—H6A	0.8600
C7—N3	1.389 (3)	Cd1—Cl2	2.4984 (8)
C7—H7A	0.9300	Cd1—Cl1	2.6283 (8)
C8—N4	1.370 (4)		
			
C2—C1—H1A	109.5	N5—C10—C9	126.8 (2)
C2—C1—H1B	109.5	N6—C10—C9	123.7 (2)
H1A—C1—H1B	109.5	C12—C11—N6	105.7 (2)
C2—C1—H1C	109.5	C12—C11—H11A	127.1
H1A—C1—H1C	109.5	N6—C11—H11A	127.1
H1B—C1—H1C	109.5	C11—C12—N5	109.9 (2)
N1—C2—N2	109.5 (2)	C11—C12—H12A	125.0
N1—C2—C1	127.2 (2)	N5—C12—H12A	125.0
N2—C2—C1	123.4 (2)	C2—N1—C3	106.5 (2)
C4—C3—N1	109.3 (2)	C2—N1—Cd1	126.89 (17)
C4—C3—H3A	125.3	C3—N1—Cd1	122.94 (17)
N1—C3—H3A	125.3	C2—N2—C4	108.6 (2)
C3—C4—N2	106.0 (2)	C2—N2—H2A	125.7
C3—C4—H4A	127.0	C4—N2—H2A	125.7
N2—C4—H4A	127.0	C6—N3—C7	106.2 (2)
C6—C5—H5A	109.5	C6—N3—Cd1	123.87 (17)
C6—C5—H5B	109.5	C7—N3—Cd1	129.69 (17)
H5A—C5—H5B	109.5	C6—N4—C8	108.8 (2)
C6—C5—H5C	109.5	C6—N4—H4B	125.6
H5A—C5—H5C	109.5	C8—N4—H4B	125.6
H5B—C5—H5C	109.5	C10—N5—C12	106.1 (2)
N3—C6—N4	109.9 (2)	C10—N5—Cd1	127.62 (17)
N3—C6—C5	126.2 (2)	C12—N5—Cd1	125.68 (16)
N4—C6—C5	123.8 (2)	C10—N6—C11	108.7 (2)
C8—C7—N3	109.3 (2)	C10—N6—H6A	125.6
C8—C7—H7A	125.4	C11—N6—H6A	125.6
N3—C7—H7A	125.4	N1—Cd1—N5	129.84 (8)
C7—C8—N4	105.7 (2)	N1—Cd1—N3	85.82 (8)
C7—C8—H8A	127.1	N5—Cd1—N3	88.17 (7)
N4—C8—H8A	127.1	N1—Cd1—Cl2	119.45 (6)
C10—C9—H9A	109.5	N5—Cd1—Cl2	110.70 (6)
C10—C9—H9B	109.5	N3—Cd1—Cl2	96.14 (6)
H9A—C9—H9B	109.5	N1—Cd1—Cl1	89.07 (6)
C10—C9—H9C	109.5	N5—Cd1—Cl1	86.50 (5)
H9A—C9—H9C	109.5	N3—Cd1—Cl1	167.67 (5)
H9B—C9—H9C	109.5	Cl2—Cd1—Cl1	96.15 (3)
N5—C10—N6	109.5 (2)		

### Hydrogen-bond geometry (Å, º)

**Table:**

*D*—H···*A*	*D*—H	H···*A*	*D*···*A*	*D*—H···*A*
N6—H6*A*···Cl1^i^	0.86	2.60	3.387 (2)	152
N4—H4*B*···Cl2^ii^	0.86	2.59	3.382 (2)	154
N2—H2*A*···Cl1^iii^	0.86	2.45	3.253 (2)	156

Symmetry codes: (i) *x*+1, *y*, *z*; (ii) −*x*+1/2, *y*−1/2, −*z*+3/2; (iii) −*x*−1/2, *y*−1/2, −*z*+3/2.
